# Impact of peritoneal metastasis on overall survival in patients with advanced endometrial cancer receiving lenvatinib plus pembrolizumab

**DOI:** 10.1007/s12672-026-04843-2

**Published:** 2026-03-15

**Authors:** Shogen Boku, Masato Kita, Tatsuki Ikoma, Tomoyo Yasuda, Nobuhiro Shibata, Hiroshi Shiraga, Yusuke Butsuhara, Hiromi Murata, Tomomi Mizokami, Hidetaka Okada, Masashi Kanai

**Affiliations:** 1 Department of Clinical Oncology, Kansai Medical University, 2-3-1, Shinmachi, Hirakata, Osaka 573-1191 Japan; 2 Department of Obstetrics and Gynecology, Kansai Medical University, Hirakata, Japan

**Keywords:** Endometrial cancer, Lenvatinib, Pembrolizumab, Starting dose, Real-world evidence, Peritoneal metastasis

## Abstract

**Purpose:**

The combination of lenvatinib plus pembrolizumab (LP) is a standard treatment for advanced or recurrent endometrial cancer. However, the optimal starting dose of lenvatinib is debated due to its toxicity profile. This study aimed to evaluate the effectiveness and safety of the LP regimen in a real-world setting and to investigate the impact of the lenvatinib starting dose on survival outcomes.

**Methods:**

We conducted a retrospective analysis of 33 patients with advanced or recurrent endometrial cancer treated with the LP regimen at Kansai Medical University Hospital between February 2022 and August 2025. We evaluated the objective response rate (ORR), progression-free survival (PFS), overall survival (OS), and safety. Prognostic factors for survival were identified using the Kaplan-Meier method and Cox proportional hazards models.

**Results:**

The median follow-up was 27.6 months. The ORR was 48.5%, with a median PFS of 6.7 months and a median OS of 21.7 months. In exploratory multivariable analysis *adjusting for age ≥ 75 years and ECOG PS ≥ 1*, a lenvatinib starting dose < 20 mg (vs. 20 mg) was associated with shorter PFS (aHR 5.83; 95% CI 1.84–18.43; p = 0.003). In contrast, peritoneal metastasis (present vs. absent) was independently associated with shorter OS (aHR 7.51; 95% CI 1.87–30.25; *p* = 0.005).

**Conclusion:**

In this real-world cohort, LP therapy was effective and tolerable. In exploratory analyses, lenvatinib starting dose and peritoneal metastasis showed distinct associations with PFS and OS, respectively; however, non-random dose selection and baseline imbalances limit causal interpretation. These findings should be considered hypothesis-generating and warrant prospective validation, particularly for patients with peritoneal metastasis.

**Supplementary Information:**

The online version contains supplementary material available at 10.1007/s12672-026-04843-2.

## Introduction

Endometrial cancer is a leading gynecologic malignancy, and the prognosis for patients with advanced or recurrent disease remains poor. The phase 3 KEYNOTE-775 trial established the combination of lenvatinib plus pembrolizumab (LP) as a standard of care, demonstrating significant improvements in both progression-free survival (PFS; HR, 0.56) and overall survival (OS; HR, 0.65) compared to chemotherapy [1]. Despite its pronounced efficacy, the LP regimen was associated with a substantial toxicity burden in the KEYNOTE-775 trial. High rates of adverse events such as hypertension, diarrhea, and hypothyroidism were reported, necessitating dose reductions or interruptions of lenvatinib in a significant proportion of patients. Consequently, the appropriateness of the recommended 20 mg/day starting dose has been debated, and whether to initiate treatment at a reduced dose remains a critical clinical question in real-world practice. In recent years, a growing body of real-world evidence has emerged to address this issue. Multi-institutional reviews and insurance claims analyses from the United States have shown that a 14 mg starting dose is predominant and that variations in the initial dose do not necessarily compromise response rates or survival outcomes [2, 3]. In contrast, other retrospective studies suggest that while a lower starting dose may reduce the frequency of dose modifications, it could be associated with inferior PFS after adjusting for factors such as age [4–6]. Systematic reviews and additional real-world studies have consistently supported the dual nature of the LP regimen—its effectiveness and notable toxicity. These studies confirm its feasibility in vulnerable populations, including older adults and patients with an ECOG performance status of 1–2 who would have been ineligible for clinical trials, but also underscore that prospective validation of dosing strategies remains an unmet need [7, 8]. In light of this background, we conducted a single-institution retrospective analysis of a real-world cohort in Japan to evaluate the effectiveness and safety of the LP regimen. The primary objective of this study was to exploratively investigate the impact of the lenvatinib starting dose on PFS and OS, thereby generating concrete hypotheses for optimizing initial dosing strategies in real-world settings, taking into account patient backgrounds such as age, sites of metastasis, and performance status.

## Methods

### Study design and patients

This study was a single-center, retrospective cohort study conducted at Kansai Medical University Hospital. We identified patients with advanced or recurrent endometrial cancer who received LP therapy between February 2022 and August 2025. Eligible patients were those who had experienced disease progression after at least one prior platinum-based chemotherapy regimen. This study was approved by the Institutional Review Board of Kansai Medical University Hospital and conducted in accordance with the Declaration of Helsinki. The requirement for informed consent was waived due to the retrospective nature of the study.

### Data collection and assessments

We retrospectively collected data from electronic medical records. The collected data included clinical and pathological characteristics, treatment history including the starting dose of lenvatinib, treatment-emergent adverse events (TEAEs), tumor response, and survival status. TEAEs were graded according to the National Cancer Institute Common Terminology Criteria for Adverse Events (CTCAE) version 5.0. Tumor response was assessed by the attending physician based on imaging studies according to the Response Evaluation Criteria in Solid Tumors (RECIST) version 1.1. The initial lenvatinib dose (20 mg or reduced dose) was determined by the treating physician based on routine clinical judgment, including age, ECOG performance status, frailty/comorbidities, baseline renal function, body size, and patient preference. Dose modifications after treatment initiation were performed according to toxicity and standard practice.

### Endpoints

The primary endpoints were progression-free survival (PFS) and overall survival (OS). PFS was defined as the time from the initiation of LP therapy to disease progression or death from any cause. OS was defined as the time from the initiation of LP therapy to death from any cause. Secondary endpoints were the objective response rate (ORR), defined as the proportion of patients with a complete or partial response, the disease control rate (DCR), defined as the proportion of patients with complete response, partial response, or stable disease, and safety.

### Statistical analysis

Patient characteristics, effectiveness, and safety data were summarized using descriptive statistics. The median follow-up time was calculated using the reverse Kaplan-Meier method. Survival curves for PFS and OS were estimated using the Kaplan-Meier method, and differences between groups were assessed using the log-rank test. Univariate and multivariate analyses for prognostic factors were performed using the Cox proportional hazards model. A P-value of < 0.05 was considered statistically significant. All statistical analyses were performed using EZR (Saitama Medical Center, Jichi Medical University, Saitama, Japan), which is a graphical user interface for R (The R Foundation for Statistical Computing, Vienna, Austria) [9]. Given the non-random selection of starting dose, multivariate Cox models were used to adjust for key clinical determinants of dose selection (age ≥ 75 years and ECOG PS ≥ 1). We additionally explored propensity score approaches as sensitivity analyses; however, strong separation and limited overlap between dose groups indicated insufficient common support, making matching/weighting estimates unstable. Therefore, we present propensity score diagnostics as supplementary material and interpret dose-group comparisons as exploratory and hypothesis-generating.

## Results

A total of 33 patients treated with lenvatinib plus pembrolizumab were included in this retrospective analysis. The median follow-up time was 27.6 months. Baseline patient and clinical characteristics are detailed in Table 1. For the overall cohort, the median age was 67.4 years, and 17 patients (51.5%) had an ECOG performance status of 0. When stratified by lenvatinib starting dose, patients in the < 20 mg group were older and had a poorer performance status compared to the 20 mg group. Specifically, all patients aged ≥ 75 years (10/10) were in the < 20 mg group, and the proportion of patients with an ECOG PS of 0 was substantially higher in the 20 mg group (81.3% vs. 23.5%). The majority of patients (78.8%) received the study treatment for recurrent disease after a median platinum-free interval of 4.0 months. Endometrioid adenocarcinoma was the most frequent histology (63.6%). Characteristics These imbalances likely reflect clinical dose selection (confounding by indication); therefore, comparisons by starting dose should be interpreted as exploratory and hypothesis-generating.

**Table 1:** Baseline patient and clinical characteristics by lenvatinib starting dose

Characteristic	All Patients (*N* = 33)	Lenvatinib 20 mg (*n* = 16)	Lenvatinib < 20 mg (*n* = 17)
Patient demographics			
Age (years), median (range)	67.4 (44.4–82.8)	60.0 (44.4–70.7)	76.7 (54.3–82.8)
≥75 years, n (%)	10 (30.3)	0 (0.0)	10 (58.8)
BMI (kg/m²), median (range)	22.8 (14.5–41.0)	27.3 (16.6–41.0)	21.8 (14.5–28.7)
ECOG Performance Status, n (%)			
0	17 (51.5)	13 (81.3)	4 (23.5)
1	15 (45.5)	3 (18.8)	12 (70.6)
2	1 (3.0)	0 (0.0)	1 (5.9)
Tumor and treatment history			
FIGO Stage at initial diagnosis, n (%)			
I	7 (21.2)	4 (25.0)	3 (17.6)
II	5 (15.2)	1 (6.3)	4 (23.5)
III	9 (27.3)	3 (18.8)	6 (35.3)
IV	12 (36.4)	8 (50.0)	4 (23.5)
Disease status at LP therapy initiation, n (%)			
Primary unresectable disease	7 (21.2)	4 (25.0)	3 (17.6)
Recurrent disease	26 (78.8)	12 (75.0)	14 (82.4)
Number of prior chemotherapy regimens, n (%)			
1	21 (63.6)	12 (75.0)	9 (52.9)
≥2	12 (36.4)	4 (25.0)	8 (47.1)
Platinum-free interval (months), median (range)	4.0 (0–71)	6.1 (1.0–28.0)	4.1 (0–71.0)
Pathological features			
Histology, n (%)			
Endometrioid adenocarcinoma	21 (63.6)	11 (68.8)	10 (58.8)
Adenocarcinoma, NOS	2 (6.1)	1 (6.3)	1 (5.9)
Dedifferentiated carcinoma	3 (9.1)	1 (6.3)	2 (11.8)
Other	7 (21.2)	3 (18.8)	4 (23.5)
MMR/MSI status, n (%)			
dMMR/MSI-high	4 (12.1)	3 (18.8)	1 (5.9)
Metastatic sites at LP therapy initiation, n (%)*			
Local/Regional	5 (15.2)	3 (18.8)	2 (11.8)
Lymph node	11 (33.3)	7 (43.8)	4 (23.5)
Peritoneum	11 (33.3)	4 (25.0)	7 (41.2)
Lung	11 (33.3)	6 (37.5)	5 (29.4)
Liver	3 (9.1)	1 (6.3)	2 (11.8)
Bone	4 (12.1)	3 (18.8)	1 (5.9)
Baseline laboratory values			
eGFR (mL/min/1.73 m²), median (range)	61.0 (20–111)	63.0 (50–103)	60.0 (20–111)
CRP (mg/dL), median (range)	0.28 (0.02–6.5)	0.33 (0.02–3.98)	0.14 (0.05–6.5)
Albumin (g/dL), median (range)	4.2 (3.2–4.7)	4.3 (3.4–4.7)	3.9 (3.2–4.6)

BMI, body mass index; CRP, C-reactive protein; dMMR, mismatch repair deficient; ECOG, Eastern Cooperative Oncology Group; eGFR, estimated glomerular filtration rate; FIGO, International Federation of Gynecology and Obstetrics; LP, lenvatinib plus pembrolizumab; MSI, microsaurikellite instability; NOS, not otherwise specified

*Patients may have more than one site of metastasis

### Treatment effectiveness

**Fig. 1:**
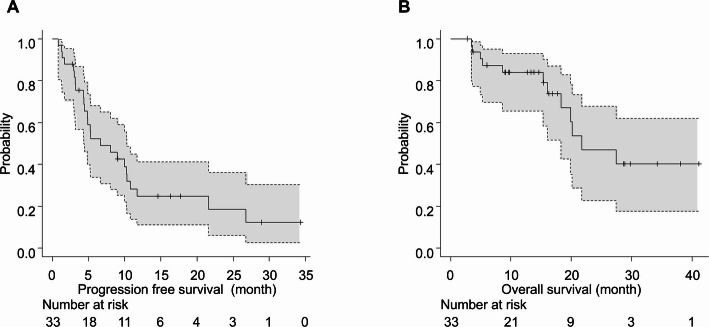
Kaplan-Meier curves for (**A**) progression-free survival and (**B**) overall survival in the entire cohort (*N* = 33). The numbers of patients at risk at each time point are shown below the x-axis

For the entire cohort, the objective response rate (ORR) was 48.5% and the disease control rate (DCR) was 87.9% (details in Supplementary Table 1). The median overall survival (OS) for the entire cohort was 21.7 months (95% CI, 18.3 months–not reached), and the median progression-free survival (PFS) was 6.7 months (95% CI, 4.4–10.3 months) (Fig. 1).

### Safety

The safety profile is detailed in Table 2. The most frequent treatment-emergent adverse events (TEAEs) of any grade were hypothyroidism (48.5%), hypertension (45.5%), and thrombocytopenia (39.4%). The most common grade ≥ 3 TEAE was thrombocytopenia (12.1%). Key immune-related adverse events (irAEs) included adrenal insufficiency (9.1%), myositis (6.1%), and pneumonitis (3.0%).

**Table 2:** Treatment-emergent adverse events

Adverse event	All grades, *n* (%)	Grade ≥ 3, *n* (%)
Hypothyroidism	16 (48.5)	0 (0.0)
Hypertension	15 (45.5)	1 (3.0)
Thrombocytopenia	13 (39.4)	4 (12.1)
Proteinuria	12 (36.4)	1 (3.0)
Decreased appetite	12 (36.4)	1 (3.0)
Fatigue/asthenia	10 (30.3)	1 (3.0)
Stomatitis/oral mucositis	8 (24.2)	2 (6.1)
Dysphonia	7 (21.2)	0 (0.0)
Peripheral neuropathy	7 (21.2)	0 (0.0)
Arthralgia	6 (18.2)	0 (0.0)
Hand-foot syndrome	6 (18.2)	0 (0.0)
Hepatic dysfunction	6 (18.2)	2 (6.1)
Nausea	6 (18.2)	0 (0.0)
Edema	6 (18.2)	0 (0.0)
Anemia	5 (15.2)	2 (6.1)
Diarrhea	5 (15.2)	0 (0.0)
Renal impairment	5 (15.2)	0 (0.0)
Rash	5 (15.2)	0 (0.0)
Adrenal insufficiency	3 (9.1)	0 (0.0)
Leukopenia	2 (6.1)	2 (6.1)
Myositis/CPK increase	2 (6.1)	1 (3.0)
Enteritis	2 (6.1)	0 (0.0)
Pneumonitis	1 (3.0)	0 (0.0)

Table lists all reported adverse events

### Prognostic factor analysis

We performed univariate and multivariate analyses to identify prognostic factors for response and survival. When stratified by the lenvatinib starting dose, the ORR was numerically higher in the 20 mg group compared to the < 20 mg group (56.3% vs. 41.2%), though the difference was not statistically significant (*p* = 0.715) (Supplementary Table 1).

### Progression-free survival

**Fig. 2:**
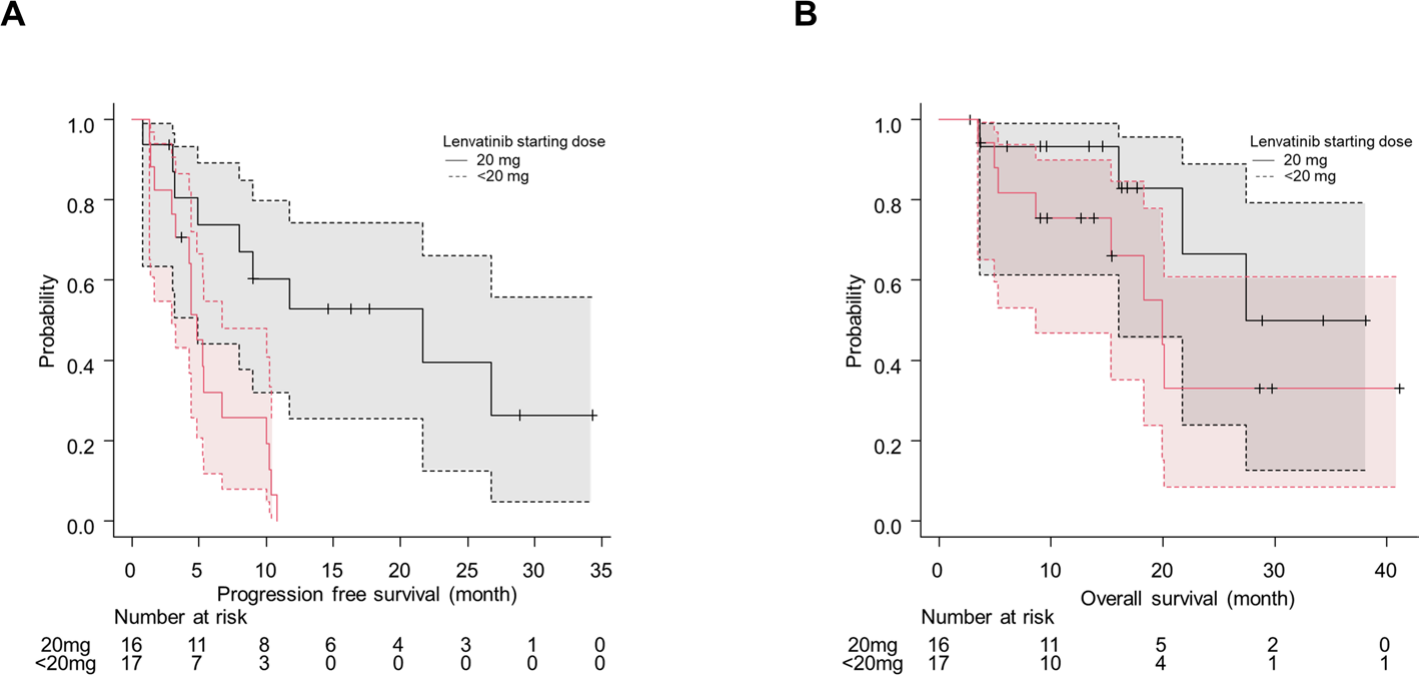
Kaplan-Meier curves for (**A**) progression-free survival and (**B**) overall survival, stratified by the starting dose of lenvatinib. The numbers of patients at risk at each time point are shown below the x-axis

In the univariate analysis, a lenvatinib starting dose of 20 mg was significantly associated with longer PFS (median 21.6 vs. 4.9 months; *p* = 0.001) (Fig. 2A). Notably, the presence of peritoneal metastasis, which was a strong predictor for OS, was not associated with PFS (*p* = 0.844). Additionally, patients aged ≥ 75 years showed a trend toward shorter PFS (*p* = 0.087). The full univariate analysis results are shown in Supplementary Table 2. To adjust for potential confounders, a multivariate analysis was performed including the starting dose and age, as age often influences the choice of starting dose. The analysis confirmed that a lenvatinib starting dose of 20 mg was the sole independent predictor of improved PFS (aHR for dose < 20 mg: 5.83, 95% CI 1.84–18.43; *p* = 0.003) (Table 3).

**Table 3:** Multivariate analysis of prognostic factors for progression-free survival

Characteristic	Adjusted hazard ratio (aHR)	95% CI	*P*-value
Lenvatinib starting dose < 20 mg (vs. 20 mg)	5.83	1.84–18.43	0.003
Age ≥ 75 years (vs. <75)	0.76	0.27–2.11	0.593
ECOG PS ≥ 1 (vs. 0)	0.79	0.34–1.86	0.589

aHR, adjusted Hazard Ratio; CI, confidence interval; ECOG PS, Eastern Cooperative Oncology Group performance status

### Overall survival

In the univariate analysis for OS, peritoneal metastasis (*p* = 0.003), lung metastasis (*p* = 0.003), and age ≥ 75 years (*p* = 0.020) were significantly associated with shorter OS (details in Supplementary Table 3). Patients starting lenvatinib at 20 mg showed a non-significant trend toward longer OS (median 27.4 vs. 19.9 months; *p* = 0.187) (Fig. 2B). In the exploratory multivariate analysis, which included clinically relevant factors, peritoneal metastasis was identified as the sole independent prognostic factor for worse OS (aHR 7.51; 95% CI, 1.87–30.25; *p* = 0.005) (Table 4).

**Table 4:** Multivariate analysis of prognostic factors for overall survival

Characteristic	Adjusted hazard ratio (aHR)	95% CI	*P*-value
Peritoneal metastasis (Present vs. Absent)	7.51	1.87–30.25	0.005
Age ≥ 75 years (vs. <75)	4.83	0.85–27.46	0.076
Lenvatinib starting dose < 20 mg (vs. 20 mg)	1.04	0.18–5.93	0.963

aHR, adjusted Hazard Ratio; CI, confidence

## Discussion

This real-world study shows that lenvatinib plus pembrolizumab (LP) achieves high disease control in advanced or recurrent endometrial cancer and suggests the starting dose of lenvatinib impacts progression-free survival (PFS). Our results support the external validity of the well-established efficacy and safety profile from the pivotal KEYNOTE-775 trial [1]. Additionally, our findings offer new insights into how initial dosing strategies affect clinical outcomes in routine practice.

Our finding that a 20 mg starting dose of lenvatinib independently predicted better PFS may be explained by the “vascular normalization window.” Anti-VEGF drugs like lenvatinib can temporarily normalize tumor blood vessels for a few days after starting treatment. This process improves oxygen levels and allows more immune cells (effector T cells) to enter the tumor [10–12]. We hypothesize that giving a full dose of lenvatinib during this short window enhances the initial priming effect of the ICI, which helps suppress early progression and leads to a better response. Preclinical data support this, showing that lenvatinib remodels the tumor microenvironment to boost the effect of anti-PD-1 antibodies [13]. Therefore, starting with a higher dose likely achieves both vascular normalization and immune priming more effectively.

The importance of sufficient drug levels early in treatment—the “quality of initial exposure”—is also supported by other studies. For instance, a Japanese multi-center study found that a relative dose intensity (RDI) of ≥ 50% in the first 8 weeks was linked to better PFS and OS, highlighting the need for adequate drug exposure early on [6]. In contrast, other real-world studies report that starting doses of 14 mg or less are common and do not show a significant loss of effectiveness in unadjusted analyses [2–4, 7]. This discrepancy highlights a key clinical challenge: balancing an effective dose with managing side effects. Our findings support a “start high, then adjust” strategy: begin with 20 mg under close monitoring and reduce the dose quickly if toxicity occurs. This approach aims to utilize the early therapeutic window while maintaining long-term treatment.

However, we must acknowledge the significant baseline imbalances between the dose groups in our cohort. As shown in Supplementary Table 4, the standardized mean differences (SMDs) for age (1.895) and ECOG PS (0.913) were substantially higher than the 0.1 threshold, indicating that the < 20 mg group was inherently more frail. While propensity score matching (PSM) could theoretically address these imbalances, our analysis of the propensity score distributions revealed very limited “common support” or overlap between the groups (Supplementary Fig. 1). In such a scenario, PSM would result in the exclusion of a large number of patients and potentially produce unstable or biased estimates. Consequently, we utilized multivariable Cox proportional hazards models as the most robust statistical approach to adjust for these key confounders (age and PS) while maximizing the available data from this real-world population.

In terms of safety, the profile of treatment-emergent adverse events was consistent with previous reports [1, 14]. The toxicity profile in our cohort underscores the necessity of proactive toxicity management [15–17] to maintain treatment feasibility.

For overall survival (OS), the strong effect of the starting dose seen for PFS was not observed. Instead, our multivariate analysis identified peritoneal metastasis as a powerful, independent predictor of worse OS. This creates a notable contrast, as peritoneal metastasis did not affect short-term outcomes like PFS in our cohort. This finding aligns with the clinical understanding that factors related to tumor biology and burden, such as the site of metastasis, are major drivers of long-term outcomes, potentially outweighing the impact of the initial dosing strategy [2–4, 6, 7]. Large-scale studies show the lung is the most common site of distant metastasis [18, 19], but peritoneal carcinomatosis generally indicates a poorer prognosis [20–22].

Our study has several limitations. As a retrospective study from a single center, it is subject to “confounding by indication”—where the physician’s choice of starting dose was influenced by the patient’s frailty. However, even after adjusting for age and PS in our multivariable model, the starting dose remained an independent predictor for PFS (aHR 5.83,* p = 0.003)*, suggesting that the dose itself has a biological impact beyond patient selection. Future prospective trials are needed to confirm these findings and determine the optimal dosing strategy for vulnerable populations.

## Conclusion

In this single-center retrospective analysis in Japan, the combination therapy of lenvatinib plus pembrolizumab demonstrated favorable effectiveness and a tolerable safety profile. While the lenvatinib starting dose was a critical predictor for progression-free survival even after adjusting for baseline frailty, our analysis revealed that the presence of peritoneal metastasis was the sole independent prognostic factor for poor overall survival. These findings suggest that while starting at 20 mg should be prioritized to maximize early disease control, LP therapy remains a relevant option that should be initiated even in patients with high-risk features like peritoneal metastasis.

## Supplementary Information

Below is the link to the electronic supplementary material.

Supplementary Material 1.

## Data Availability

The datasets generated during and/or analysed during the current study are available from the corresponding author on reasonable request.
