# Supplementary material for: Impact of peritoneal metastasis on overall survival in patients with advanced endometrial cancer receiving lenvatinib plus pembrolizumab
Source: Discov Oncol. 2026 Mar 15;17:624. doi: 10.1007/s12672-026-04843-2 (PMC13103040; doi:10.1007/s12672-026-04843-2)

**Supplementary Material**

**Supplementary Table 1: Treatment Efficacy and Outcomes by Lenvatinib Starting Dose**

| **Efficacy Endpoint** | **Total (N=33)** | **Lenvatinib <20 mg (n=17)** | **Lenvatinib**  **20 mg (n=16)** | **P-value** |
| --- | --- | --- | --- | --- |
| **Response Rates, n (%)** |  |  |  |  |
| Objective Response Rate | 16 (48.5) | 7 (41.2) | 9 (56.3) | 0.715 |
| Complete Response | 3 (9.1) | 1 (5.9) | 2 (12.5) |  |
| Partial Response | 13 (39.4) | 6 (35.3) | 7 (43.8) |  |
| Disease Control Rate¹ | 29 (87.9) | 14 (82.4) | 15 (93.8) | 0.344 |

Abbreviations: CI, confidence interval; DCR, disease control rate; NE, not estimable; ORR, objective response rate.

¹Disease control rate includes patients with complete response, partial response, and stable disease.

P-values for response rates were calculated using Fisher's exact test.

**Supplementary Table 2: Univariate Analysis of Prognostic Factors for Progression-Free Survival**

| **Characteristic** | **Category** | **N** | **Median PFS (months) (95% CI)** | **P-value** |
| --- | --- | --- | --- | --- |
| **Lenvatinib Starting Dose** | **20 mg** | **16** | **21.6 (4.9–NE)** | **0.001** |
|  | **<20 mg** | **17** | **4.9 (3.0–6.7)** |  |
| Age | ≥75 years | 10 | 5.3 (1.3–10.3) | 0.087 |
|  | <75 years | 23 | 9.0 (4.3–21.6) |  |
| ECOG PS | ≥1 | 16 | 5.3 (3.2–10.8) | 0.557 |
|  | 0 | 17 | 9.0 (3.2–26.8) |  |
| Platinum-Free Interval | ≥6 months | 14 | 8.5 (4.3–26.8) | 0.312 |
|  | <6 months | 19 | 4.9 (3.2–10.8) |  |
| Number of prior regimens | ≥2 | 12 | 5.3 (3.0–NE) | 0.399 |
|  | 1 | 21 | 8.0 (4.4–11.7) |  |
| Bone Metastasis | Present | 4 | 3.8 (1.3–NE) | 0.252 |
|  | Absent | 29 | 8.0 (4.9–10.8) |  |
| Lung Metastasis | Present | 14 | 9.0 (3.0–NE) | 0.377 |
|  | Absent | 19 | 6.7 (4.4–10.3) |  |
| Liver Metastasis | Present | 3 | 8.0 (3.2–NE) | 0.408 |
|  | Absent | 30 | 6.7 (4.4–10.8) |  |
| Lymph Node Metastasis | Present | 14 | 9.0 (3.2–21.6) | 0.489 |
|  | Absent | 19 | 5.3 (3.2–10.8) |  |
| Peritoneal Metastasis | Present | 14 | 6.7 (4.4–10.8) | 0.844 |
|  | Absent | 19 | 9.0 (3.2–11.7) |  |

Abbreviations: CI, confidence interval; ECOG PS, Eastern Cooperative Oncology Group performance status; NE, not estimable; PFS, progression-free survival

**Supplementary Table 3: Univariate Analysis of Prognostic Factors for Overall Survival**

| **Characteristic** | **Category** | **N** | **Median OS (months) (95% CI)** | **P-value** |
| --- | --- | --- | --- | --- |
| **Peritoneal Metastasis** | **Present** | **14** | **16.0 (5.3–NE)** | **0.003** |
|  | **Absent** | **19** | **Not Reached (18.3–NE)** |  |
| **Lung Metastasis** | **Absent** | **19** | **19.9 (8.6–21.7)** | **0.003** |
|  | **Present** | **14** | **Not Reached (15.3–NE)** |  |
| **Age** | **≥75 years** | **10** | **18.3 (3.5–NE)** | **0.020** |
|  | **<75 years** | **23** | **Not Reached (20.1–NE)** |  |
| ECOG PS | ≥1 | 16 | Not Reached (8.6–NE) | 0.636 |
|  | 0 | 17 | 21.7 (16.0–NE) |  |
| Lenvatinib Starting Dose | 20 mg | 16 | 27.4 (16.0–NE) | 0.187 |
|  | <20 mg | 17 | 19.9 (8.6–NE) |  |
| Platinum-Free Interval | ≥6 months | 14 | Not Reached (15.3–NE) | 0.174 |
|  | <6 months | 19 | 20.1 (5.3–NE) |  |
| Lymph Node Metastasis | Present | 14 | 21.7 (16.0–NE) | 0.355 |
|  | Absent | 19 | 20.1 (8.6–NE) |  |
| Liver Metastasis | Present | 3 | 18.9 (16.0–NE) | 0.435 |
|  | Absent | 30 | 27.4 (18.3–NE) |  |
| Bone Metastasis | Present | 4 | 5.3 (4.9–NE) | 0.979 |
|  | Absent | 29 | 21.7 (18.3–NE) |  |

**Abbreviations:** CI, confidence interval; ECOG PS, Eastern Cooperative Oncology Group performance status

Supplementary Table 4: Standardized mean differences (SMDs) for key baseline variables by starting dose

SMDs are shown as <20 mg vs 20 mg; values >0.10 indicate meaningful imbalance.

| Variable | SMD (<20 vs 20) |
| --- | --- |
| Age (years) | 1.895 |
| Age ≥75 years | 1.291 |
| ECOG PS ≥1 | 0.913 |
| BMI (kg/m2) | -0.56 |
| Albumin (g/dL) | -0.781 |
| eGFR (mL/min/1.73 m2) | -0.21 |
| CRP (mg/dL) | 0.199 |
| Number of prior regimens | 0.769 |
| Peritoneal metastasis | 0.439 |

**Supplementary Figure 1: Propensity score distribution for starting dose <20 mg by dose group**


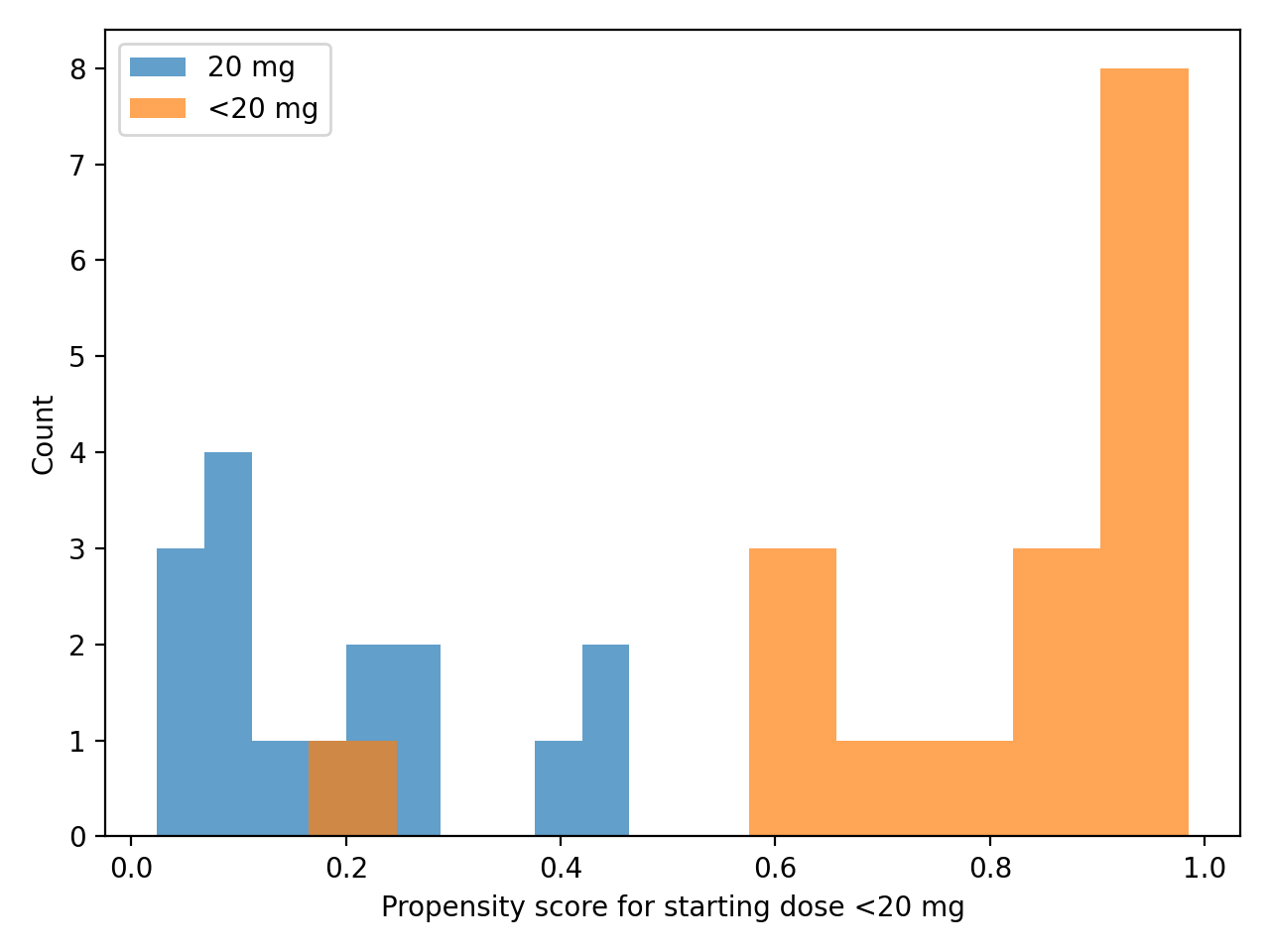

Supplement: Supplementary file 1 — Supplementary Material 1. [file 12672_2026_4843_MOESM1_ESM.docx]
